# Vision-related quality of life and psychosocial well-being of patients with episcleritis and scleritis: a neglected essence?

**DOI:** 10.1186/s12348-021-00265-z

**Published:** 2021-09-22

**Authors:** Apurva Ratna Tamrakar, Ranju Kharel Sitaula, Sagun Narayan Joshi, Manjita Bajracharya

**Affiliations:** 1 Phaco and Vitreo-Retinal Surgeon, Kathmandu Eye Centre, Patan, Nepal; 2 Department of Ophthalmology, Maharajgunj Medical Campus, Institute of Medicine, Tribhuvan University, B. P. Koirala Lions Centre for Ophthalmic Studies, Maharajgunj, Kathmandu, Nepal; 3 Department of General practice and emergency medicine, Tribhuvan University Teaching Hospital, Kathmandu, Nepal

**Keywords:** Corticosteroids, Episcleritis, Quality of life, Scleritis, Visual function

## Abstract

**Background:**

To assess the change in vision-related quality of life and psychosocial well-being of the patients with episcleritis and scleritis patients before and after treatment.

**Results:**

This one-and-a-half-year prospective study was conducted among 76 eyes of 71 new patients of episcleritis and scleritis. A structured questionnaire was used to assess the visual and to analyze the change in effect size. The male-to-female ratio was 1:1.536. Episcleritis was seen in 41 cases (57.7%) while scleritis was seen in 30 cases (42.3%). Patients with episcleritis had statistically significant improvement in general function score (GF) (*p* < 0.05) using paired t-test. The effect size showed medium improvement (approximately 0.5). Whereas there was no statistically significant change in psychosocial impact (PI), visual symptoms (VS) scoring, and a total score (p < 0.05) using paired t-test. The effect size showed no improvement for PI and total score and small improvement for VS score. Patients with scleritis had statistically significant improvement in general function score (GF), visual symptoms (VS) scoring and total score (*p* < 0.05) using paired-t-test. The effect size showed medium improvement (approximately 0.5) for general function score (GF) and total score. However, the effect size showed only a small improvement (approximately 0.2) for psychosocial impact (PI) score.

**Conclusions:**

VisionRelated Quality of Life of patients with scleritis showed significant improvement following treatment unlike episcleritis indicating scleritis more adversely affecting psychosocial well-being.

## Background

Scleral inflammation is seen in 1 in every 6000 new patients [1]. In Nepal, the prevalence of scleritis is documented to be 0.03% [2]. Though, less common, the consequence of an incorrect diagnosis or inappropriate treatment cannot only to blindness but can also have an important impact on general and psychosocial well-being. It is therefore important that all ophthalmologists are aware of what constitutes serious disease needing urgent treatment, and what can safely be left alone possibly without any treatment at all. This is relatively easy when the inflammation affects the anterior sclera but is much more difficult when the posterior segment is involved.

Episcleritis is a mild, non-vision-threatening inflammation of the episclera that may recur over irregular intervals for many years. It is highly essential to recognize its benign nature and not to induce vision-threatening complications, by over treating episodes of episcleritis like steroid induced cataract and glaucoma.

Scleritis particularly causes vision loss via its complications or treatment-related complications due to its chronicity and frequent relapses. The anterior segment complications of anterior scleritis include keratitis, uveitis, cataract, glaucoma, and cystoid macular oedema may develop in patients with uveitis associated with anterior scleritis. Posterior scleritis is associated with more vision-threatening complications like serous retinal detachment, optic disc oedema, choroidal effusion, macular oedema, and retinal vasculitis [1].

Due to its frequent recurrences and chronic course, particularly for scleritis, it is associated with significant stress, even during inactive phases. This may even affect daily and work-related activities which may ultimately lead to anxiety and depression. This may prompt a diminished visual-related quality of life (VR-QOL).

Quality of life (QOL) is defined by World Health Organization (WHO) as an “individual’s perception of their position in life in the context of the culture and value systems in which they live and in relation to their goals, expectations, standards, and concerns” [3]^.^ Here in our context vision-related as well as health-related QOL are pertinent. Hence, the purpose of this study was to evaluate the change in visual function among cases with episcleritis and scleritis before and after treatment. This study is the first of its kind from Nepal, where importance has been given to identifying the change in visual function and its impact on daily living activities, evaluated in ophthalmic diseases.

## Methods

A prospective observational hospital-based study was conducted among the episcleritis and scleritis cases at the uveitis clinic of B. P Koirala Lions Centre for Ophthalmic Studies, the eye department of the Institute of Medicine between January 2015 and June 2016. A total of 71 new patients were recruited from the general and uveitis clinic over 18 months and were followed up for 4 weeks. All participants went through an extensive ocular examination and filled up the vision function questionnaire. The Indian Vision Function Questionnaire (IND-VFQ) [2], designed in 2005 to survey visual function in a populace of visually impaired and blind people living in a low-income nations, was adopted. Testing of the IND-VFQ for reliability (Cronbach’s alpha > 0.70), validity, and consistency indicated that it is appropriate for use in clinical research [2]. The questionnaire was given to all the patients before starting the treatment, by an interviewer (one of the investigator) and the patients repeated the same questionnaire, administered by the same interviewer, at the 4th week of treatment. The IND-VFQ, which consisted of 33 items in three sections [4]. The first section had 21-items for general function, the second section had 5-items for psychosocial impact and the third section consisted of 7-items for visual symptoms. The items in the general function section covered mobility, household performance, economic activity, and activities of daily living. The psychosocial scales had items concerning social, family, and personal wellbeing. The visual symptoms had a scale for items, such as vision, photophobia, and glare. A four-point response scale assessed visual symptoms and psychosocial impact from 1 (best score) to 4 (worst score). The general functioning questions had a 5-point scale, from 1 (best score) to 5 (worst score) [2]. For each scale, a composite score was calculated as a cumulative total of individual responses expressed as a percentage of the maximum score possible and then transformed, such that 100 represented the best possible score (no difficulty with any of the items in that scale) and 0 the worst score (maximum difficulty in that scale) [5]. The responses of patients for each question were transformed to a “0 to 100” scale using a standard scoring algorithm and uniformity for result calculation maintained among the point score of visual symptoms, psychosocial impact, and general function impact.

Each patient underwent a complete ophthalmic evaluation and necessary investigations were done, the findings were recorded, and treatment was administered as clinically indicated. Sociodemographic data were also collected.

Data were entered into SPSS Statistics version 20. A *p*-value of < 0.05 was considered statistically significant. Paired t-test and Effect size (ES) analysis were carried comparing the pre and post-treatment scores to work out the differences in VR-QOL following treatment of episcleritis and scleritis. The ES was defined as the mean change in VFQ score at follow-up, divided by its standard deviation (SD) at baseline. The ES reflects the magnitude of change in VFQ in response to treatment commenced at baseline. Cohen defines an ES of 0.2 as a small change, 0.5 as a medium change, and ≥ 0.8 as a large change [6]. Approval from the ethical committee of the institutional review board was obtained and adherence to the tenets of the Declaration of Helsinki was maintained. Written consent was obtained from the participants before the ocular examination and administration of the questionnaire.

## Results

Out of 71 enrolled patients (scleritis + episcleritis) in total, nearly 2/3rd patients (60.6%) were females. Thus the male to female ratio was 1:1.53. Among them, 57.7% (41 cases) had episcleritis while the rest 42.3% (30 cases) had scleritis. In the episcleritis group, the mean age of the patient was 33.88 ± 11.63 years, with the youngest being 16 years old and the eldest being 55 years of age. Among the scleritis group, the mean age of the patients was 36.77 ± 10.35 years. The youngest case was of 23 years old and the oldest being 63 years of age. Unilateral involvement was seen in 38 patients (92.7%) with episcleritis and 28 patients (93.3%) with scleritis.

**Fig. 1:**
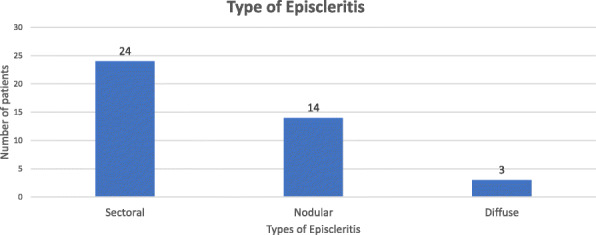
Bar diagram showing types of Episcleritis

Among 41 episcleritis patients, sectoral episcleritis was seen in 24 cases (58.5%), whereas nodular episcleritis was seen in 14 cases (34.2%), followed by diffuse episcleritis in 3 cases (7.3%) (Fig. 1).

**Fig. 2:**
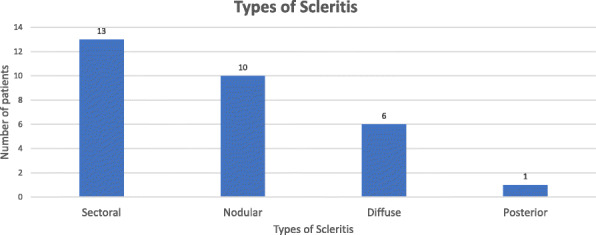
Bar diagram showing types of scleritis

Among 30 scleritis cases, there were 29 cases (97%) of anterior scleritis and only 1 case (3%) of posterior scleritis. Sectoral scleritis was seen in 13 cases (43.3%), whereas nodular scleritis was seen in 10 cases (33.3%), followed by diffuse scleritis in 6 cases (20.0%) as shown in Fig. 2. However, there were no cases of isolated necrotizing scleritis seen within the duration of our study period.

The visual function was assessed in episcleritis and scleritis patients on presentation and at the 4th week of treatment in terms of general function (GF), psychosocial impact (PI), and visual symptoms (VS). All three parameters showed statistically significant improvement (*p* < 0.0001) in visual function after treatment. The effect size showed a change in all scales (approx. 0.5).

Patients with episcleritis had statistically significant improvement in general function score (GF) (*p* < 0.05) using paired t-test. The effect size showed medium improvement (approximately 0.5). Whereas there was no statistically significant change in psychosocial impact (PI), visual symptoms (VS) scoring and the total score (*p* < 0.05) using paired t-test. The effect size showed no improvement for PI and total score and small improvement for VS score (Table 1).

**Table 1:** Comparison of Vision-Related Quality of life following treatment for episcleritis

Scale	Pre Treatment		Post Treatment		Paired differences		95% Confidence Interval of the Difference		*P*-value	Effect size
	Mean	Standard Deviation	Mean	Standard Deviation	Mean	Standard Deviation	Lower	Upper		
GF	22.71	2.261	22.07	1.808	0.634	0.888	0.354	0.914	**< 0.001**	0.714
PI	5.68	1.312	5.66	1.755	0.024	0.689	−0.193	0.242	0.822	0.035
VS	8.02	1.782	7.78	1.754	0.244	1.067	−0.093	0.581	0.151	0.229
Total	36.34	4.252	35.85	4.845	0.488	2.461	−0.289	1.265	0.212	−1.973

Patients with scleritis had statistically significant improvement in general function score (GF), visual symptoms (VS) scoring and the total score (*p* < 0.05) using paired t-test. The effect size showed medium improvement (approximately 0.5) for general function score (GF) and total score. However, the effect size showed only a small improvement (approximately 0.2) for psychosocial impact (PI) score. The details are shown in Table 2.

**Table 2:** Comparison of Vision-Related Quality of life following treatment for scleritis

Scale	Pre Treatment		Post Treatment		Paired differences		95% Confidence Interval of the Difference		*P*-value	Effect size
	Mean	Standard Deviation	Mean	Standard Deviation	Mean	Standard Deviation	Lower	Upper		
GF	27.57	9.365	24.87	7.960	2.700	4.970	0.844	4.556	**0.006**	0.543
PI	8.07	2.947	7.27	3.205	0.800	3.056	−0.341	1.8941	0.162	0.262
VS	10.60	4.065	9.47	3.431	1.133	2.700	0.125	2.142	**0.029**	0.419
Total	46.10	14.466	41.63	13.574	4.467	9.493	0.922	8.011	**0.015**	0.470

## Discussion

Both episcleritis and scleritis affect individuals in a multitude of ways. While the clinical symptoms the patients suffer are always catered to, their general well-being and the psychological impacts caused by the disease entity is frequently neglected. These impacts are however equally essential to be noted and analyzed for a complete assessment of patient well-being.

Widely used clinical measures of vision provide information regarding the disease process but they may not capture all the important aspects of visual function from the patient’s perspective and the effect of treatment on the person as a whole [7]. Other researches regarding vision function assessment have shown issues with recognizing individuals/faces (both near and distance), with mobility (walking/running, driving, using public transport), reading, and light-related sensitivity issues, such as glare and difficulties with night driving [8, 9]. Perceptions of vision in a variety of diseases are now used in clinical trials to evaluate the efficacy of medical or surgical interventions.

Herein, for the first time in Nepal, the vision-related quality of life was assessed in the Nepalese episcleritis and scleritis patients in terms of general function (GF), psychosocial impact (PI) and visual symptoms (VS). And we found that the patients with episcleritis had statistically significant improvement in GF score (*p* < 0.05) using paired t-test. The effect size showed medium improvement (approximately 0.5). Whereas there was no statistically significant change in PI score, VS scoring and the total score (*p* < 0.05) using paired t-test. The effect size showed no improvement for PI and total score and small improvement for VS score.

Patients with scleritis had significant improvement in GF score, VS scoring and the total score (*p* < 0.05). The effect size showed medium improvement (approximately 0.5) for GF score and total score. However, the effect size showed only a small improvement (approximately 0.2) for PI score. Though PI score showed improvement following treatment but the change was not statistically significant (*p* > 0.05). This could be related to the fear in patients regarding recurrences in the future and gradual improvement throughout treatment.

Hoeksema and Los et al. [10] reported lower scores on vision-specific social functioning, vision-specific mental health, vision-specific role difficulties, and vision-specific dependency in patients undergoing treatment for uveitis or related ophthalmic complications. This implies these patients are more stressed and disappointed by their vision and they require more help given their visual perception [10].

Arvind V et al. in 2008 they reported significant improvement in VR-QOL (Vision-Related Quality of life) in all scales following treatment for uveitis (*p* < 0.001) [5]. Another similar study by Gamal et al. in 2016 in the similar setting concluded anterior uveitis and posterior uveitis patients had significant improvement (*p* ≤ 0.001) in all 3 scales but panuveitis and intermediate uveitis had significant improvement (*p* < 0.05) in only 2 scales [11]. The effect size showed small to large change in all 3 scales. However, there were no available past literature based upon changes in visual functions and quality of life among episcleritis and scleritis cases of Nepal.

Niemeyer et al. concluded using IND-VFQ scores, on average, over its 6 months study period (*p* = 0.0001) [12]. They observed a decreased mental component summary score (*p* = 0.04) and decreased vitality subscale (*p* = 0.001), while the SF-36 physical component summary score did not significantly differ throughout the trial. Although uveitis treatment was related to better vision and vision-related personal satisfaction, patient-reported physical health showed no change throughout a half year of treatment, with decreased psychological wellness.

Sugar et al. [13] conducted a similar longitudinal study comparing individuals with non-infectious uveitis treated with fluocinolone acetonide implant with those treated with systemic corticosteroid. Patients in both treatment groups displayed comparative improvement in NEI-VFQ-25 scores after 3 years of follow-up. People with worst visual acuity and visual fields at first were observed to be related to lower NEI-VFQ-25 scores for both treatment categories.

Kempen et al. had evaluated the risks along with quality of life outcomes associated with fluocinolone acetonide implant versus systemic therapy with corticosteroids and immunosuppression in cases of intermediate, posterior and panuveitis. This in its entirety was a part of the Multicenter Uveitis Steroid Treatment (MUST) trial and follow-up Study [14]. Self-detailed QOL measures at first supported implant-treated patients. Later with time, the QOL measures for both the groups were assessed and narrowed down to a favourable score.

Kaleemunnisha et al. [7] reported a greater effect size along with statistically significant betterment in the composite score, at follow-up, (*p* = 0.004; effect size = 1.03). They observed improvement in the QOL scores corresponding to reduced inflammation and improved visual acuity, with the initiation of immunosuppressives.

Such a study of quantitatively measuring the impact of the disease and treatment using vision-related quality of life has not yet been done in patients of episcleritis and scleritis. Visual acuity is generally not affected in typical episcleritis and scleritis, but it may be affected when they are associated with keratitis, uveitis as well as in posterior scleritis with macular oedema or exudative retinal detachment. Psychological problems emerged as a concern with longstanding redness which may or may not be associated with pain and the fear of multiple recurrences.

Vision-Related Quality of Life of patients with scleritis showed significant improvement following treatment unlike episcleritis indicating scleritis more adversely affecting psychosocial well-being.

## Conclusion

In conclusion, the universal aim of eye care is to improve the QOL of visually impaired people. This study aims to show that resolution of episcleritis and scleritis after treatment restores associated adverse effects on the quality of life in terms of general functioning, psychosocial impact and visual function. Thus, assessment of visual function in episcleritis and, to a greater extent, in cases of scleritis before and after treatment can be considered an important instrument to evaluate the presumed benefit of treatment. Early diagnosis and prompt treatment of episcleritis and scleritis is not only important for vision but also to improve vision-related quality of life.

## Limitations

The main limitation of the study was the time period of follow up of the patients. It needs longerperiod to evaluate the complete outcome and other long-term complications of episcleritis andmore importantly scleritis which in turn affect their quality of life.

## Data Availability

Not applicable.
